# Prognostic models versus single risk factor approach in first‐trimester selective screening for gestational diabetes mellitus: a prospective population‐based multicentre cohort study

**DOI:** 10.1111/1471-0528.16446

**Published:** 2020-09-01

**Authors:** F van Hoorn, MPH Koster, CA Naaktgeboren, F Groenendaal, A Kwee, M Lamain‐de Ruiter, A Franx, MN Bekker

**Affiliations:** ^1^ Department of Obstetrics and Gynaecology University Medical Centre Utrecht Utrecht University Utrecht The Netherlands; ^2^ Department of Obstetrics and Gynaecology Erasmus MC University Medical Centre Rotterdam Rotterdam The Netherlands; ^3^ Julius Centre for Health Sciences and Primary Care University Medical Centre Utrecht Utrecht The Netherlands; ^4^ Department of Neonatology University Medical Centre Utrecht Utrecht University Utrecht The Netherlands

**Keywords:** Biomarkers, clinical prediction rule, diabetes in pregnancy, glucose, impact analysis, obstetrics, perinatal complications

## Abstract

**Objectives:**

To evaluate whether (1) first‐trimester prognostic models for gestational diabetes mellitus (GDM) outperform the currently used single risk factor approach, and (2) a first‐trimester random venous glucose measurement improves model performance.

**Design:**

Prospective population‐based multicentre cohort.

**Setting:**

Thirty‐one independent midwifery practices and six hospitals in the Netherlands.

**Population:**

Women recruited before 14 weeks of gestation without pre‐existing diabetes.

**Methods:**

The single risk factor approach (presence of at least one risk factor: BMI ≥30 kg/m^2^, previous macrosomia, history of GDM, positive first‐degree family history of diabetes, non‐western ethnicity) was compared with the four best performing models in our previously published external validation study (Gabbay‐Benziv 2014, Nanda 2011, Teede 2011, van Leeuwen 2010) with and without the addition of glucose.

**Main outcome measures:**

Discrimination was assessed by *c*‐statistics, calibration by calibration plots, added value of glucose by the likelihood ratio chi‐square test, net benefit by decision curve analysis and reclassification by reclassification plots.

**Results:**

Of the 3723 women included, a total of 181 (4.9%) developed GDM. The *c*‐statistics of the prognostic models were higher, ranging from 0.74 to 0.78 without glucose and from 0.78 to 0.80 with glucose, compared with the single risk factor approach (0.72). Models showed adequate calibration, and yielded a higher net benefit than the single risk factor approach for most threshold probabilities. Teede 2011 performed best in the reclassification analysis.

**Conclusions:**

First‐trimester prognostic models seem to outperform the currently used single risk factor approach in screening for GDM, particularly when glucose was added as a predictor.

**Tweetable abstract:**

Prognostic models seem to outperform the currently used single risk factor approach in screening for gestational diabetes.

## Tweetable abstract

Prognostic models seem to outperform the currently used single risk factor approach in screening for gestational diabetes.

## Introduction

Alongside the worldwide obesity epidemic, the incidence of gestational diabetes mellitus (GDM) is rising and currently affects 5% of all pregnancies in Europe and 1–42% worldwide depending on the studied population and the applied diagnostic criteria.^1^, ^2^ Short‐term complications of GDM include pre‐eclampsia, large‐for‐gestational‐age neonates and perinatal death.^3^, ^4^ Moreover, women with GDM and their offspring both have an increased risk to develop obesity, type 2 diabetes mellitus and cardiovascular disease later in life.^5^, ^6^, ^7^ Early diagnosis and management of GDM enable treatment and could improve pregnancy outcomes.^8^, ^9^, ^10^ Therefore, in most countries, testing for GDM by means of an oral glucose tolerance test (OGTT) in the second or third trimester of pregnancy is part of standard obstetric care.^11^

Testing can be performed universally in all women, or selectively in women with one or more prespecified risk factors for GDM (single risk factor approach).^11^, ^12^, ^13^ In low‐risk populations, many women without GDM are subjected to a burdensome OGTT, thereby stressing healthcare budgets and logistics, but on the other hand, women with GDM could be missed with selective testing. Therefore, improvement in the accuracy of identification of high‐risk women for GDM is warranted.

First‐trimester prognostic regression models, in which generally available clinical predictors are weighted and combined, are an alternative and more personalised approach to identify high‐risk women compared with the currently used single risk factor approach. These first‐trimester models are not yet incorporated in clinical guidelines, despite their availability and good predictive performance after external validation,^14^ because there is limited evidence for whether screening with these models indeed improves current risk‐factor‐based selective testing later in pregnancy.^15^

The aim of this study was to compare the predictive performance of the four best performing first‐trimester prognostic models for GDM in a previously conducted external validation study^14^ (Gabbay‐Benziv 2014,^16^ Nanda 2011,^17^ Teede 2011^18^ and van Leeuwen 2010^19^) with the currently used single risk factor approach in a general low‐risk obstetric population. Blood biomarkers could improve the predictions of these models, which only contain clinical predictors, but testing for them is invasive and potentially costly. This does not account for random venous glucose, which is already routinely measured in the first trimester of pregnancy to screen for pre‐existing diabetes mellitus in our setting.^20^ Therefore, we also explored whether the addition of a first‐trimester glucose assessment would further improve prognostic model performance.

## Methods

### Study population and design

Analyses were performed on data from a population‐based prospective multicentre study (Risk EStimation for PrEgnancy Complications to provide Tailored care; RESPECT). The cohort was primarily used for the external validation of first‐trimester prognostic models for GDM and pre‐eclampsia.^14^, ^21^ Between December 2012 and January 2014, a total of 3736 women with a singleton pregnancy were included before 14 weeks of gestation in 31 independent midwifery practices, five regional hospitals and one tertiary referral hospital in the Netherlands. Women with pre‐existing diabetes mellitus were excluded from the analysis. The study was approved by the medical ethics committee of the University Medical Centre Utrecht (protocol no. 12‐432/C) on 6 September 2012. Written informed consent was obtained from all participants.

### Predictors and baseline characteristics

Baseline characteristics, including predictors, were all measured in the first trimester of pregnancy through a set of standardised questionnaires issued to both pregnant women and obstetric staff. Demographics provided by the women included age (years), height (centimetres), ethnicity (Caucasian, African, Asian, mixed, other), smoking (yes/no), first‐degree family history of diabetes mellitus (yes/no), parity (number of previous pregnancies beyond 16 weeks of gestation), method of conception (spontaneous, ovulation drugs, in vitro fertilisation), history of GDM (yes/no), history of macrosomia >90th centile (yes/no) and level of education (low/medium/high).^22^, ^23^ The obstetric healthcare professional recorded the woman's weight (kilograms), blood pressure (mmHg), first‐trimester random venous glucose (mmol/l) and gestational age (based on a crown–rump length measurement at ultrasound examination).^24^ Body mass index (BMI) was calculated as weight in kilograms divided by the squared height in metres.

### Reference method

According to Dutch clinical guidelines, women were considered at high‐risk for GDM if they had one or more prespecified risk factors: BMI >30 kg/m^2^, previous child with a birthweight above the 95th centile or 4500 g, history of GDM, first‐degree family member with any type of diabetes mellitus, non‐western ethnicity with a high prevalence of diabetes mellitus (Hindustani, Moroccan, Turkish, Middle Eastern, Asian), presence of polycystic ovary syndrome and/or a history of unexplained intrauterine fetal death.^20^ Polycystic ovary syndrome and a history of unexplained intrauterine fetal death were not available in the RESPECT cohort so could not be included in the reference method for analysis.

### Prognostic models

The four best performing first‐trimester prognostic models in the external validation study^14^ were used for current analysis: Gabbay‐Benziv 2014,^16^ Nanda 2011,^17^ Teede 2011^18^ and van Leeuwen 2010.^19^ Predictors included in all models were: ethnicity, BMI and history of GDM. Maternal age was incorporated in all, except van Leeuwen 2010. Family history of diabetes was included by Teede 2011 and van Leeuwen 2010. Nanda 2011 and van Leeuwen 2010 used parity. Only Nanda 2011 included a history of macrosomia and systolic blood pressure was solely included by Gabbay‐Benziv 2014. The full equations of the models are provided in the Supplementary material (Table S1).

### Outcome

Pregnancy outcomes were collected by obstetric staff by filling in a Case Report Form after delivery. The presence of GDM was recorded as well as the need for insulin therapy to optimise glucose regulation. All women received obstetric care according to Dutch clinical guidelines for screening and diagnosis of GDM.^20^ According to this guideline, GDM is diagnosed when a 75‐g 2‐hour OGTT results in either a fasting glucose level of ≥7.0 mmol/l (126 mg/dl) or a post‐load glucose level of ≥7.8 mmol/l (140 mg/dl).^20^, ^25^ All women who were considered high‐risk for GDM by the reference method in the first trimester underwent testing for GDM with an OGTT between 24 and 28 weeks of gestation. Furthermore, at any point in pregnancy, women with signs or symptoms of GDM, e.g. macrosomia or polyhydramnios, underwent an OGTT; regardless of whether they were considered high‐risk for GDM or not. With this strategy we presume that we detected most women with GDM. However, GDM could have been missed because universal testing was not performed. These false‐negatives could hypothetically have been classified correctly as high‐risk by the selected prognostic models, thereby underestimating their performance leading to an increased risk of falsely not rejecting the null hypothesis (type II error).

Neonatal outcomes included sex (male/female), birthweight (grams) and birthweight centile (based on national reference curves adjusted for parity, gestational age, sex and ethnicity^26^).

An applicable core outcome set from the CROWN database was not available for this study.

### Statistical analysis

All analyses were performed on the multiple imputed data set with ten imputations that was also used for the external validation study using the same set of inclusion criteria (see Supplementary material, Table S2).^14^ Imputed values were included when calculating descriptive statistics. Analyses were performed on each of the imputed data sets and results were pooled by applying Rubin's rules without any transformation of the estimates.^27^

The models were recalibrated by fitting logistic regression models using the linear predictor as the only covariate, resulting in an updated calibration slope and intercept.^14^, ^28^ The performance of the reference method and recalibrated prognostic models before and after the addition of glucose was assessed. Because of a skewed distribution, a natural log transformation of glucose was applied. Discrimination of the models was described by *c*‐statistics, showing the ability to distinguish women who did and did not develop GDM. The added value of glucose was assessed using the likelihood ratio chi‐square test. Calibration plots of the models were conducted by plotting all ten imputed data sets as if they were one large data set, showing the agreement between predicted probabilities and observed cases.

The net benefit of the updated prognostic models at different threshold probabilities was compared in a decision curve analysis. The net benefit is defined as the proportion of false positives (high‐risk women without GDM) subtracted from the proportion of true positives (high‐risk women with GDM) at a certain cut‐off risk.^29^

To further compare the reference method with the updated prognostic models, we evaluated model performance in two scenarios. In scenario A we explored whether models identified more women with GDM while classifying the same number of women as high‐risk: model sensitivity and specificity were calculated when the proportion of women classified as high‐risk was held constant at 29% (i.e. the proportion of women classified as high‐risk by the reference method). In scenario B, we explored whether models classified fewer women as high‐risk while identifying the same number of women with GDM: the proportion of women considered high‐risk by the model and specificity were calculated when the sensitivity was held constant at 71% (i.e. the sensitivity of the reference method).

The best performing model for scenarios A and B was further compared with the reference method in a reclassification plot. This plot shows how the proportion of women with and without GDM, stratified by risk for GDM, are classified by the prognostic model compared with the reference method.

Statistical analyses were performed by the mice and rms packages of R‐3.5.1 for Windows (http://cran.r‐project.org). Results are reported according to TRIPOD guidelines for prediction models.^30^

### Public and patient involvement

A Dutch patient confederation for patients who had a pregnancy complicated by hypertensive disorders (HELLP foundation), was involved in defining the main research question and the design of the RESPECT study. Furthermore, a qualitative study was undertaken to explore pregnant women's perceptions, preferences and needs regarding prediction models for first‐trimester screening for common pregnancy complications.^31^ The final results of this study will be disseminated through regional obstetric collaboration associations, and will be made publicly accessible on the websites of collaborating partners.

### Funding

The RESPECT study was conducted with the support of the Netherlands Organisation for Health Research and Development (project no. 209020004). The funding source had no role in the design, conduct, analyses or reporting of the study or in the decision to submit the manuscript for publication.

## Results

### Study population

Women from the RESPECT cohort with pre‐existent diabetes mellitus were excluded (*n* = 13).^14^ The mean age of the 3723 women included for analysis was 30.8 (SD 4.2) years and 1655 (44.5%) of them were nulliparous (Table 1). Median prepregnancy BMI was 23.2 kg/m^2^ (interquartile range 21.1–26.2 kg/m^2^) and the majority of the study population was of Caucasian origin (*n* = 3387, 91.0%). A history of GDM or of a large‐for‐gestational‐age neonate was present in 59 (2.9%) and 230 (11.1%) multiparous women, respectively. In the current pregnancy, GDM was diagnosed in 181 (4.9%) women.

**Table 1:** Characteristics and pregnancy outcomes of women in the RESPECT cohort

	RESPECT cohort (*n* = 3723)
**Characteristic**	
Age (years)	30.8 (4.2)
Body mass index (prepregnancy), (kg/m^2^)	23.2 (21.1–26.2)
Body mass index (first trimester), (kg/m^2^)	23.7 (21.5–26.7)
Systolic blood pressure (mmHg)	115 (12)
Diastolic blood pressure (mm Hg)	67 (8)
First‐trimester random venous glucose (mmol/l)	4.7 (4.4–5.1)
Ethnicity	
Caucasian	3387 (91.0)
African	30 (0.8)
Asian	53 (1.4)
Mixed	77 (2.1)
Other	176 (4.7)
Education	
Low	270 (7.3)
Medium	1273 (34.29)
High	2180 (58.6)
Smoking during pregnancy	334 (9.0)
Family history of diabetes mellitus	543 (14.6)
Method of conception	
Spontaneous	3429 (92.9)
Ovulation drugs	99 (2.7)
In vitro fertilisation	110 (3.0)
Nulliparous	1655 (44.5)
History of gestational diabetes mellitus	59 (1.6)
History of macrosomia (>90th centile)	230 (6.2)
**Pregnancy outcomes**	
Gestational diabetes mellitus	181 (4.9)
Insulin‐dependent	33 (0.9)
Gestational age at delivery (days)	280 (273–285)
Sex (male)	1902 (51.1)
Birthweight (g)	3520 (3190–3880)
Centile	55 (30–79)
>90th centile	494 (13.3)

Data are number (%), mean (standard deviation) or median (interquartile range). This table was adapted from Lamain‐de Ruiter et al.^14^ and includes imputed values where there were missing values.

### Reference method

The reference method, reflecting care‐as‐usual, classified 1083 (29.1%) of the population as high‐risk for GDM. The sensitivity, specificity and the positive and negative predictive values of the reference method were 0.71 (95% CI 0.64–0.78), 0.73 (95% CI 0.72–0.75), 0.12 (95% CI 0.10–0.14) and 0.98 (95% CI 0.97–0.99), respectively. The *c*‐statistic of the reference method was 0.72 (95% CI 0.68–0.76) (Table 2).

**Table 2:** *C*‐statistics for the reference method and the four first‐trimester prognostic models for GDM before and after the addition of the new predictor first‐trimester random venous glucose

	Before addition of glucose	After addition of glucose	Improvement*
	*c*‐statistic (95% CI)	*c*‐statistic (95% CI)	*P*‐value
Reference^20^	0.72 (0.68–0.76)	–	
Gabbay‐Benziv 2014^16^	0.75 (0.71–0.79)	0.78 (0.75–0.82)	0.16
Nanda 2011^17^	0.78 (0.74–0.82)	0.80 (0.76–0.83)	0.20
Teede 2011^18^	0.77 (0.73–0.81)	0.80 (0.76–0.84)	0.16
van Leeuwen 2010^19^	0.74 (0.71–0.78)	0.78 (0.74–0.81)	0.14

CI, confidence interval; GDM, gestational diabetes mellitus.

* Improvement of model fit after addition of glucose (likelihood ratio test).

### Prognostic models with and without the addition of the new predictor glucose

The *c*‐statistics of the recalibrated prognostic models ranged from 0.74 to 0.78 (Table 2). The discrimination of all four prognostic models improved after addition of the new predictor glucose, illustrated by *c*‐statistics ranging from 0.78 to 0.80; however these increases were not statistically significant according to the likelihood ratio test (Table 2). All models showed adequate calibration (see Supplementary material, Figure S1). The calibration plots of Nanda 2011 and Teede 2011 showed sporadic underestimation or overestimation, but this improved after the models were updated with glucose.

### Reference method compared with updated prognostic models for GDM

**Figure 1:**
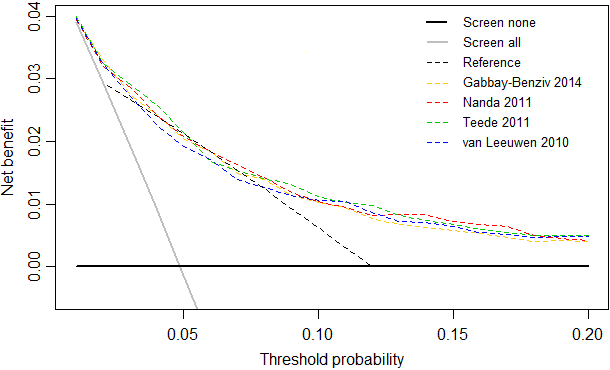
Decision curves analysis for the reference method and the four updated first‐trimester prognostic models for GDM. GDM, gestational diabetes mellitus.

The decision curve analysis showed that the reference method has a higher net benefit between a threshold probability of 2% and 12% compared with testing all or none of the population (Figure 1). The updated prognostic models had a higher net benefit than the reference method for most threshold probabilities. The curves of the updated prognostic models were situated close together, indicating that the net benefit of the models among different thresholds was comparable. The model with the highest net benefit differed per threshold, with Teede 2011 or Nanda 2011 most often being the most favourable model.

**Figure 2:**
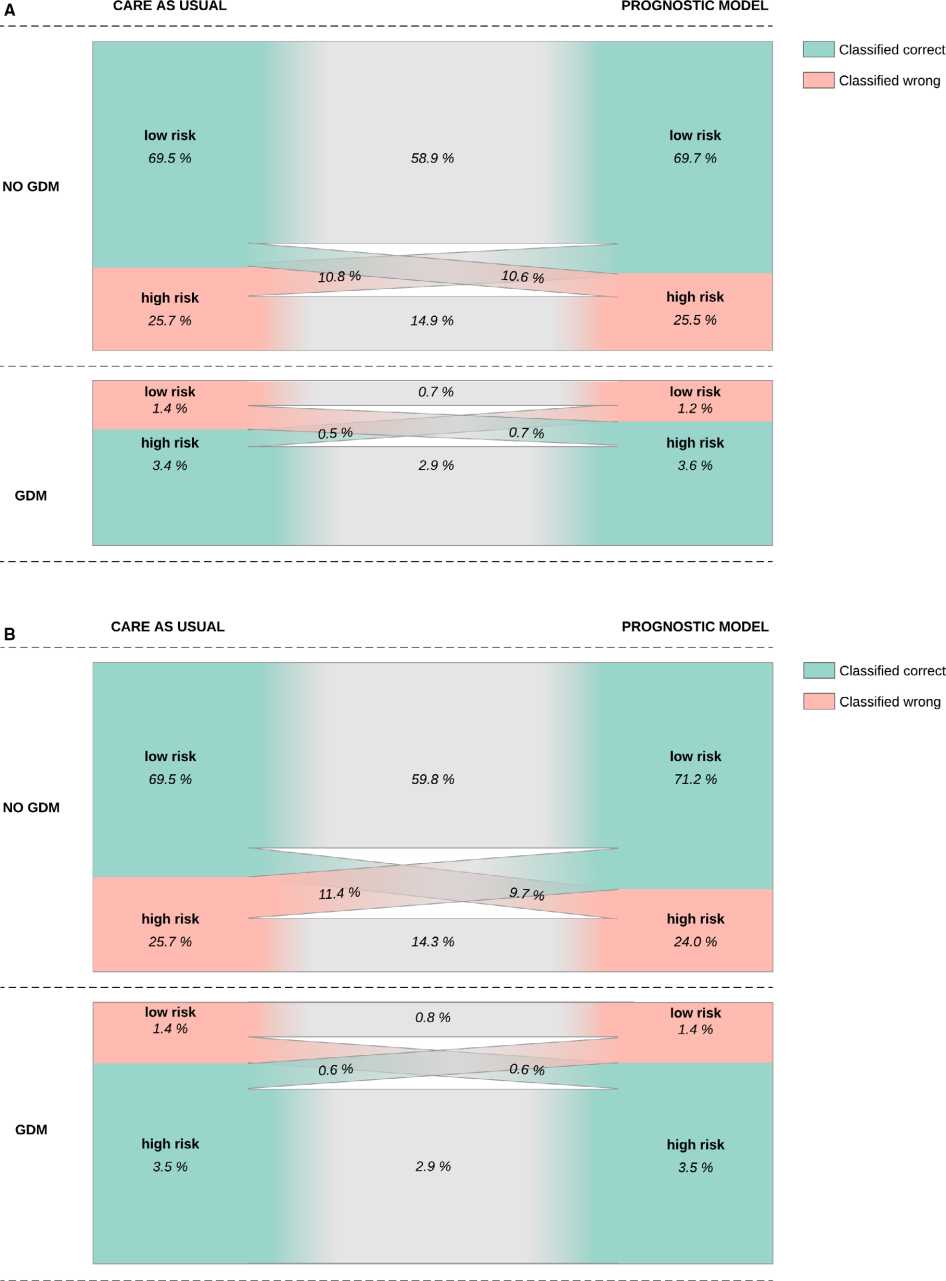
Reclassification plots comparing the reference method with the Teede 2011 model updated with first‐trimester random venous glucose for scenarios A (A) and B (B). (A) Scenario A: the proportion of women considered high‐risk was held constant at 29% (i.e. the proportion of women classified high‐risk by the reference method). (B) Scenario B: the sensitivity was held constant at 71% (i.e. the sensitivity of the reference method).

The updated prognostic models were compared with the reference method in two scenarios where Teede 2011 performed best in both (see Supplementary material, Table S3). In scenario A, the sensitivity of the updated prognostic models ranged from 69% to 74% compared with 71% for the reference method, while the proportion of high‐risk women was held constant at 29% (i.e. the proportion of women classified high‐risk by the reference method) (see Supplementary material, Table S3). The accuracy of the updated Teede model was better compared with the reference method, reflected by 5 (0.2%) more women with GDM being classified as high‐risk and 5 (0.2%) fewer women without GDM being defined as high‐risk (Figure 2A). In scenario B, the proportion of women classified as high‐risk by the updated prognostic models ranged from 27% to 33% compared with 29% by the reference method, while the sensitivity was held constant at 71% (i.e. the sensitivity of the reference method) (see Supplementary material, Table S3). The updated Teede model detected as many women with GDM as with the reference method by screening 17 (2%) fewer women (Figure 2B).

## Discussion

### Main findings

This study showed that first‐trimester prognostic models for GDM seem to outperform a reference method based on the presence of one or more prespecified risk factors. All four investigated prognostic models yielded higher discrimination than the reference method, illustrated by *c*‐statistics of 0.74–0.78 compared with 0.72. The performance of the prognostic models further improved consistently, although not significantly, after addition of the predictor first‐trimester random venous glucose (*c*‐statistic 0.78–0.80). Overall, the model of Teede 2011 with added glucose performed best in our cohort. Selective testing for GDM might be made more efficient after screening with first‐trimester prognostic models, because a lower proportion of false positives (i.e. high‐risk without GDM) would be subjected to an OGTT, avoiding unnecessary healthcare costs and testing burden for women.

### Strengths and limitations

One of the strengths of this study is the large prospective population‐based cohort of unselected pregnant women, which is the preferred design for model updating and comparison studies.^32^ Additionally, missing data were adequately handled by multiple imputation to minimise bias. The reference method in this study reflects care‐as‐usual in the Netherlands; however, similar risk factors are recommended in the internationally used guideline of the National Institute for Health and Care Excellence (NICE).

Limitations include that our population was predominantly low‐risk for GDM (i.e. predominantly Caucasian, normal BMI, normotensive, medium to high educational level), which may make our findings less generalisable to more high‐risk or otherwise distinct populations. However, these alterations in predictive performance might not be extensive because of recalibration since the Teede 2011 model, that performed best in our cohort, was developed in an Australian population (*c*‐statistic 0.70) with a higher prevalence of overweight or obesity, non‐Caucasian ethnicity and GDM.^18^ Another limitation is that universal testing for GDM was not applied in the cohort. GDM cases that were missed by the single risk factor approach could have been correctly classified as high‐risk by prognostic models, thereby underestimating their performance in this study. Also, two risk factors used in Dutch care‐as‐usual (polycystic ovary syndrome, unexplained fetal demise) were not available in the data set. We calculated that this could have led to 0.8–1.1% of women being misclassified as low risk instead of high risk at the most (data not shown) and we therefore assume that the influence of these missing variables on prognostic performances was limited.

### Interpretation

Selective risk‐factor‐based strategies have been evaluated in various populations, however, to our knowledge, only one previous study by Syngelaki et al.^15^ compared first‐trimester prognostic models for GDM with a reference method based on the presence of one or more risk factors. They showed a higher detection rate for a new prognostic model, but not for the externally validated prognostic models (including van Leeuwen 2010,^19^ Teede 2011^18^ and Nanda 2011^17^) compared with the reference method.^15^ They used the same diagnostic criteria for GDM and their reference method was similar except for a history of macrosomia, which was defined as ≥4500 g by the NICE – compared with >4500 g or >95th birthweight centile in our study.^20^, ^33^ As the single risk factor approach is not a regression model that can be recalibrated to match the disease prevalence and predictor distribution in a population, this contradictory finding may be explained by differences in study population, especially regarding risk factors for GDM; women were more often of non‐Caucasian origin and had a higher BMI than in our cohort.

Consistent with our results, Harrison et al.^34^, ^35^ and Abell et al.^34^, ^35^ confirmed the incremental value of first‐trimester venous glucose when added to the Teede 2011 model; although, they evaluated fasting measurements in a smaller sample of high‐risk women and used different diagnostic criteria for GDM. Sweeting et al.^36^ also found higher first‐trimester glucose levels in women with GDM compared with controls, but did not include glucose in their final model. Reported discrimination for first‐trimester glucose only was similar to our findings, with *c*‐statistics ranging from 0.58 to 0.73 in literature compared with 0.68 (95% CI 0.65–0.72) in our study.^37^, ^38^, ^39^, ^40^, ^41^, ^42^, ^43^

Risk factors are embedded in most international guidelines for GDM and these could all potentially benefit by replacing their risk‐factor‐based approach with a more efficient prognostic model.^13^ In countries were glucose is not routinely measured in the first trimester of pregnancy, implementation of a prognostic model for GDM can still be considered, as most prognostic models without the addition of glucose seem to outperform the reference method as well. Most European guidelines, e.g. NICE guidelines, recommend selective risk‐factor‐based testing between 24 and 28 weeks of gestation; testing in early pregnancy is only performed in women with a history of GDM.^13^, ^20^, ^33^, ^44^ Other international guidelines, e.g. the American Diabetes Association and the Australasian Diabetes in Pregnancy Society, use risk factors to determine which women should be tested for pre‐existent diabetes in early pregnancy; but recommend universal testing in the second or early third trimester.^13^, ^45^, ^46^ Based on our results, we are not proposing to avert universal testing for GDM in high‐risk populations. Although prognostic models may also be considered in those high‐risk areas when models are able to improve the selection of women at risk for GDM more considerably in the future. Prognostic models may also be of use by identifying women who could benefit the most from preventive measures; although, clear recommendations on GDM prevention in clinical practice are not yet stated.^47^, ^48^, ^49^, ^50^ Whether prognostic models improve risk‐factor‐based testing for diabetes in early pregnancy could also be evaluated.

Unfortunately, some women with GDM are still missed by selective testing because they do not have any of the known risk factors and the majority of high‐risk women do not develop GDM. Future studies should therefore investigate the incremental value of new predictors,^51^ e.g. biomarkers such as adiponectin^35^, ^52^, ^53^, ^54^ or maternal visceral fat measurements,^55^ and should, in particular, focus on increasing specificity and the false‐negative group. Contrary to maternal characteristics, these potential predictors are not readily available and their clinical applicability should be investigated including the perspectives of pregnant women and obstetric healthcare professionals on acceptability, cost‐effectiveness and other implementation outcomes.^51^, ^56^ Furthermore, future research should focus on risk communication and should identify barriers and facilitators to understand and improve the implementation process, as well as, evaluate the effect of a prognostic model on decision‐making and whether this improves both pregnancy outcomes and utilisation of healthcare resources (impact analysis).^51^, ^56^, ^57^

## Conclusion

To conclude, in this study we showed that four first‐trimester prognostic models for GDM seem to outperform a method solely based on the presence of one or more risk factors. These models have the potential to improve the efficiency of selective testing for GDM and to decrease the number of women undergoing an unnecessary and burdensome OGTT. In turn, this will likely improve identification and treatment of women with GDM, healthcare expenditure, and maternal and child health. The investigated prognostic models consist of readily available predictors and could therefore easily be implemented in clinical practice. Although generalisability should be examined before implementation in more high‐risk or otherwise distinct populations to account for differences in disease prevalence and predictor distribution. Barriers and facilitators for implementation in clinical practice should be determined in an implementation study.

### Disclosure of interests

The authors declare no competing interests. Completed disclosure of interests forms are available to view online as supporting information.

### Contribution to authorship

AF, AK, FG, MLR, MPHK and the RESPECT study group were involved in the RESPECT study design and acquisition of data. CAN, FH, MNB and MPHK were responsible for the current study concept. CAN and FH performed data‐analysis. FH, MNB and MPHK were involved in the initial interpretation of data and drafting of the manuscript. All authors (AF, AK, CAN, FG, FH, MLR, MNB, MPHK) were responsible for interpretation of data and critical revision of the manuscript. All authors agreed on the final version to be published. All authors had full access to the data (including statistical reports and tables) in the study and take responsibility for the integrity of the data and the accuracy of the data analysis. The guarantors (AF, MNB) accept full responsibility for the work and/or the conduct of the study, had access to the data and controlled the decision to publish. The corresponding author attests that all listed authors meet the authorship criteria and that no others meeting the criteria have been omitted.

### Details of ethics approval

The RESPECT study was approved by the medical ethics committee of the University Medical Centre Utrecht on 6 September 2012 (protocol no. 12‐432/C), and was performed in line with the principles of the Declaration of Helsinki. Written informed consent was obtained from all participants.

### Funding

The RESPECT study was conducted with the support of the Netherlands Organisation for Health Research and Development (project no 209020004). The funding source had no role in the design, conduct, analyses or reporting of the study or in the decision to submit the manuscript for publication.

### Data sharing

De‐identified patient data set and statistical code are available from the corresponding author.

## Supporting information


**Figure S1.** Calibration plots of the four first‐trimester prognostic models for GDM before and after the addition of the new predictor first‐trimester random venous glucose.

**Table S1.** Full equations of the original, recalibrated and updated models.

**Table S2.** Baseline characteristics of patients in the RESPECT cohort, stratified by variables that were available for imputation including the quantification of the amount of missing data.

**Table S3.** Updated prognostic models compared with the reference method in two scenarios.

Supplementary Material

Supplementary Material

Supplementary Material

Supplementary Material

Supplementary Material

Supplementary Material

Supplementary Material

Supplementary Material
