# Supplementary material for: Prognostic models versus single risk factor approach in first‐trimester selective screening for gestational diabetes mellitus: a prospective population‐based multicentre cohort study
Source: BJOG. 2020 Sep 1;128(4):645–54. doi: 10.1111/1471-0528.16446 (PMC7891327; doi:10.1111/1471-0528.16446)
Supplement: Supplementary file 1 — Figure S1. Calibration plots of the four first‐trimester prognostic models for GDM before and after the addition of the new predictor first‐trimester random venous glucose. [file BJO-128-645-s001.pdf]

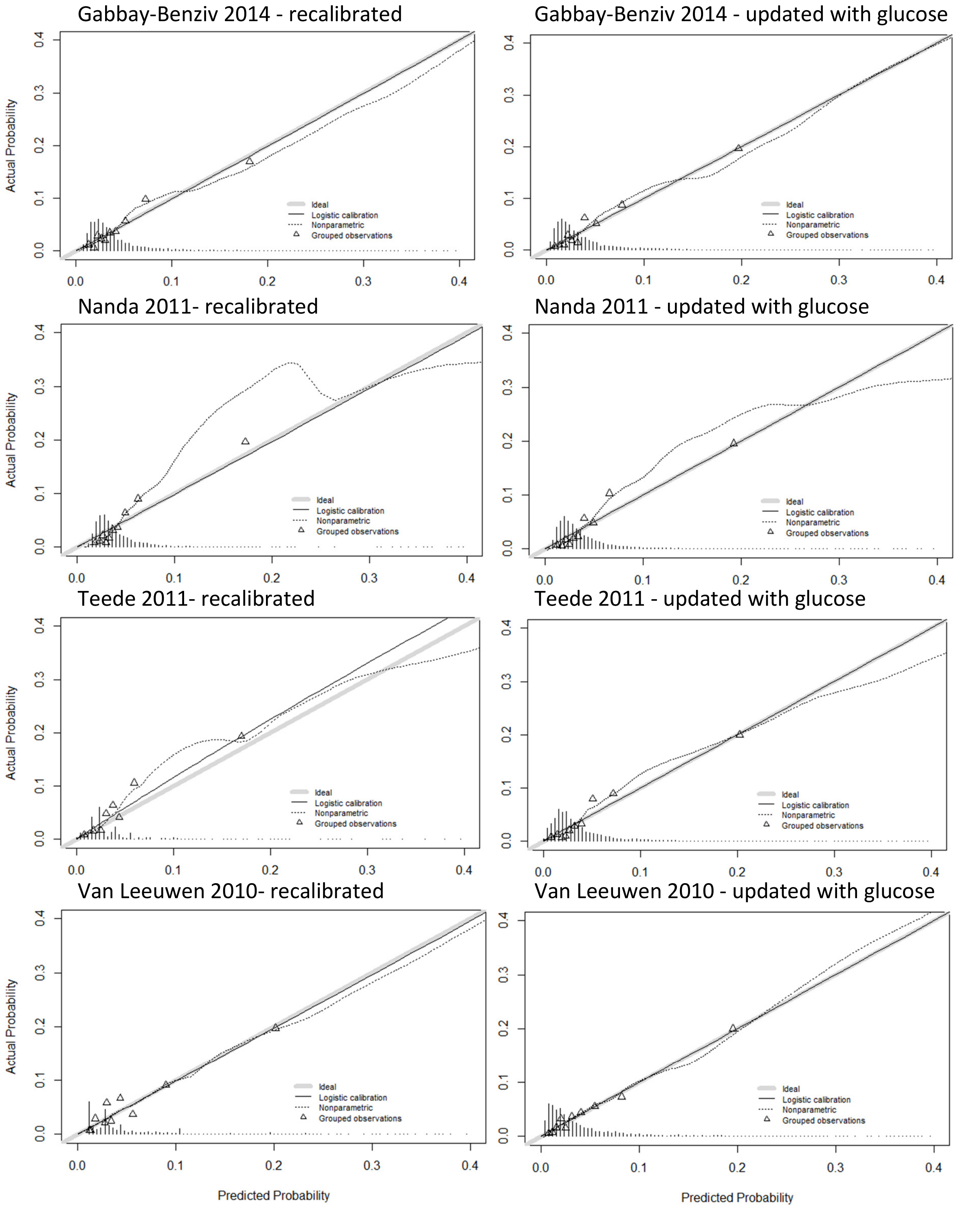

Figure S1. Calibration plots of the four first trimester prognostic models for GDM before and after the addition of the new predictor glucose.
