# Supplementary material for: Prognostic models versus single risk factor approach in first‐trimester selective screening for gestational diabetes mellitus: a prospective population‐based multicentre cohort study
Source: BJOG. 2020 Sep 1;128(4):645–54. doi: 10.1111/1471-0528.16446 (PMC7891327; doi:10.1111/1471-0528.16446)
Supplement: Supplementary file 2 — Table S1. Full equations of the original, recalibrated and updated models. [file BJO-128-645-s002.pdf]

Table S1. Full equations of the original, recalibrated, and updated models. The probability of developing gestational diabetes was calculated using logistic regression. The probability of GDM = e^X/(1+e^X) where X is the linear predictor.

Name	Original model	Recalibrated model	Updated model
Reference 2010[20]	$X_{original}$ =presence of at least one of the following factors[a]:<br>▪ history of GDM<br>▪ BMI > 30 (kg/m2) at first perinatal control<br>▪ history of macrosomia (>4500g)<br>▪ first degree family member with diabetes<br>▪ high risk ethnicities (Southern Asians, Hindu, Afro-Caribbean, or from the Middle East, Morocco, or Egypt)	NA	NA
Gabbay-Benziv 2014[16]	$X_{original}$ = -11.569 + 0.064 (age, yrs) + 0 (if white race) + 2.026 (if Asian race) + 0.083 (if African race) + 1.661 (if other nonwhite race) + 2.144 (history of GDM) + 0.034 (systolic blood pressure, mmHg) + 0.082 (BMI, kg/m2)	$X_{recalibrated}$ = -0.209 + 0.867 ($X_{original}$)	$X_{glucose\ added}$= 0.004 + 0.054 ($X_{original)}$ + 0.152 (ln(glucose))
Nanda 2011[17]	$X_{original}$ = α + 0.058 (age, yrs) + 0.113 (BMI, kg/m2) + 0 (if Caucasian ethnicity) + 0.888 (if Asian ethnicity) + 0 (nulliparous) + 3.723 (if parous with previous GDM) + 0.67 (parous with previous LGA above 90th percentile)	$X_{recalibrated}$ = -7.071 + 0.824 ($X_{original}$)	$X_{glucose\ added}$= -0.500 + 0.071 ($X_{original)}$ + 0.139 (ln(glucose))
Teede 2010[18]	$X_{original}$ = α + 0 (if age <25 yrs) + 0.92 (if age 25-29 yrs) + 1.22 (if age 30-34 yrs) + 1.69 (if age 35-39 yrs) + 1.95 (if age ≥40 yrs) + 0 (if BMI <20 kg/m2) + 0.53 (if BMI 20.0-24.9 kg/m2) + 0.69 (if BMI 25.0-26.9 kg/m2) + 0.83 (if BMI 27.0-29.9 kg/m2) + 1.28 (if BMI 30.0-34.9 kg/m2) + 1.82 (if BMI ≥35.0 kg/m2) + 1.31 (if Asian race) + 0.06 (if African race) + 0.37 (if other race) + 0.53 (if family history of DM, first degree) + 2.39 (if history of GDM)	$X_{recalibrated}$ = -5.606 + 1.132 ($X_{original}$)	$X_{glucose\ added}$= -0.319 + 0.071 ($X_{original)}$ + 0.152 (ln(glucose))
van Leeuwen 2010[19]	$X_{original}$ = -6.1 + 0.83 (if non-Caucasian race) + 0.57 (if first degree family history of DM) – 0.67 (if parous without history of GDM) + 0.5 (if parous with history of GDM) + 0.13 (BMI in kg/m2 with BMI <22 transformed to 22, if >30 transformed to 30)	$X_{recalibrated}$ = 0.752 + 1.314 ($X_{original}$)	$X_{glucose\ added}$= 0.019+ 0.070 ($X_{original)}$ + 0.160 (ln(glucose))

[a] Current practice also classifies women with a history of unexplained IUFD or PCOS as high-risk, but these predictors were not available in the cohort. *IUFD*, intra uterine fetal demise. *yrs*, years. *BMI*, body mass index. *DM*, diabetes mellitus. *LGA*, large-for-gestational-age. *kg*, kilograms. *m^2*, squared meters. *GDM*, gestational diabetes mellitus. *glucose,* first trimester random venous glucose. *gr*, grams. *IVF*, in vitro fertilization.
