# Supplementary material for: Prognostic models versus single risk factor approach in first‐trimester selective screening for gestational diabetes mellitus: a prospective population‐based multicentre cohort study
Source: BJOG. 2020 Sep 1;128(4):645–54. doi: 10.1111/1471-0528.16446 (PMC7891327; doi:10.1111/1471-0528.16446)
Supplement: Supplementary file 3 — Table S2. Baseline characteristics of patients in the RESPECT cohort, stratified by variables that were available for imputation including the quantification of the amount of missing data. [file BJO-128-645-s003.pdf]

**Table S2.** Baseline characteristics of patients in the RESPECT cohort, stratified by variables that were available for imputation

| Characteristic | Patients with missing information | Complete cases (n=2603) | Patients with at least one missing value (n=1120) | P | Overall RESPECT cohort (n=3723)* |
|---|---|---|---|---|---|
| Age (years) | 168 (4.5) | 30.9 (4.34)† | 30.7 (3.91)† | 0.29 | 30.8 (4.2)† |
| Body mass index, before pregnancy | 46 (1.2) | 23.3 (21.2-26.3)† | 23.0 (20.9-25.9)† | 0.01‡ | 23.2 (21.1-26.2)† |
| Body mass index | 182 (4.8) | 23.8 (21.6-26.9)† | 23.4 (21.3-26.3)† | 0.01‡ | 23.7 (21.5-26.7)† |
| Systolic blood pressure (mm Hg) | 65 (1.7) | 114 (12)† | 115 (12)† | 0.48 | 115 (12)† |
| Diastolic blood pressure (mm Hg) | 64 (1.7) | 67 (8)† | 67 (8)† | 0.09 | 67 (8)† |
| Glucose (mmol/L) | 171 (4.5) | 4.7 (4.3-5.1)† | 4.7 (4.4-5.1)† | 0.34 | 4.7 (4.4-5.1)† |
| Ethnicity | | | | | |
| White | | 1665 (89.0) | 1066 (95.2) | | 3387 (91.0) |
| African | | 17 (0.9) | 2 (0.2) | | 30 (0.8) |
| Asian | 732 (19.7) | 30 (1.6) | 11 (1.0) | <0.001‡ | 53 (1.4) |
| Mixed | | 44 (2.4) | 15 (1.3) | | 77 (2.1) |
| Other | | 115 (4.4) | 26 (2.3) | | 176 (4.7) |
| Education | | | | | |
| Low | | 198 (7.6) | 52 (4.6) | | 270 (7.3) |
| Middle | 223 (6.0) | 825 (31.7) | 362 (32.3) | 0.004‡ | 1273 (34.29) |
| High | | 1357 (57.0) | 706 (63.0) | | 2180 (58.6) |
| Smoking during pregnancy | 0 | 258 (9.9) | 73 (6.5) | 0.001‡ | 334 (9.0) |
| History of chronic hypertension | 1 (0.0) | 43 (1.7) | 14 (1.2) | 0.44 | 57 (1.5) |
| Family history of diabetes mellitus | 1 (0.0) | 389 (15.0) | 154 (13.8) | 0.37 | 543 (14.6) |
| Method of conception | | | | | |
| Spontaneous | | 2396 (93.1) | 1033 (92.2) | | 3429 (92.9) |
| Ovulation drugs | 30 (0.8) | 61 (2.4) | 38 (3.4) | 0.20 | 99 (2.7) |
| In vitro fertilisation | | 82 (3.2) | 28 (2.5) | | 110 (3.0) |
| Nulliparous | 4 (0.0) | 1143 (44.0) | 509 (45.4) | 0.43 | 1655 (44.5) |
| History of gestational diabetes mellitus | 0 | 47 (1.8) | 12 (1.1) | 0.13 | 59 (1.6) |
| History of macrosomia (>90th percentile) | 0 | 146 (5.6) | 84 (7.5) | 0.03‡ | 230 (6.2) |
| Recurrent miscarriages (≥2) | 4 (0.0) | 173 (6.7) | 59 (5.3) | 0.13 | 232 (6.2) |
| History of fetal death | 0 | 58 (2.2) | 16 (1.4) | 0.14 | 74 (2.0) |
| Gestational diabetes mellitus in pregnancy | 263 (7.0) | 116 (5.0) | 53 (4.7) | 0.84 | 181 (4.9) |
| Insulin dependent | | 20 (0.8) | 13 (1.2) | 0.33 | 33 (0.9) |
| Gestational age at delivery (days) | 342 (9.2) | 280 (273-285)† | 280 (274-286)† | 0.36 | 280 (273-285)† |
| Sex (male) | 358 (9.6) | 1154 (50.7) | 569 (52.3) | 0.40 | 1902 (51.1) |
| Birthweight (g) | | 3504 (3200-3860)† | 3540 (3216-3880)† | 0.13 | 3520 (3190-3880)† |
| Percentile | 372 (10.0) | 55 (30-77)† | 57 (32-80)† | 0.07 | 55 (30-79)† |
| >90th percentile | | 256 (12.0) | 140 (13.2) | 0.37 | 494 (13.3) |

Data are no (%) unless stated otherwise. *Data in column includes imputed data for those with missing values. †Data are mean (standard deviation) or median (interquartile range). ‡Significant at the P<0.05 level. Adopted from the external validation study previously published by our group (Lamain-de Ruiter M et al. BMJ. 2016;354:i4338).
