# Supplementary material for: Prognostic models versus single risk factor approach in first‐trimester selective screening for gestational diabetes mellitus: a prospective population‐based multicentre cohort study
Source: BJOG. 2020 Sep 1;128(4):645–54. doi: 10.1111/1471-0528.16446 (PMC7891327; doi:10.1111/1471-0528.16446)
Supplement: Supplementary file 4 — Table S3. Updated prognostic models compared with the reference method in two scenarios. [file BJO-128-645-s004.pdf]

Table S3. Updated prognostic models compared to the reference method in two scenarios

	Scenario A[a]		Scenario B[b]	
	**sensitivity (%)**	**specificity (%)**	**high-risk (%)**	**specificity (%)**
Reference [20]	*71*	*73*	*29*	*73*
Gabbay-Benziv 2014 [16]	69	73	31	71
Nanda 2011 [17]	71	73	29	73
Teede 2011 [18]	74	73	27	75
van Leeuwen 2010 [19]	68	73	33	69

[a] Scenario A: model sensitivity and specificity were calculated when the proportion of women considered high-risk was held constant at 29% (i.e. the proportion of women classified high-risk by the reference method).

[b] Scenario B: the proportion of women classified high-risk by the model and specificity were calculated when the sensitivity was held constant at 71% (i.e. the sensitivity of the reference method).
